# Culturing of Cardiac Fibroblasts in Engineered Heart Matrix Reduces Myofibroblast Differentiation but Maintains Their Response to Cyclic Stretch and Transforming Growth Factor β1

**DOI:** 10.3390/bioengineering9100551

**Published:** 2022-10-14

**Authors:** Meike C. Ploeg, Chantal Munts, Tayeba Seddiqi, Tim J. L. ten Brink, Jonathan Breemhaar, Lorenzo Moroni, Frits. W. Prinzen, Frans. A. van Nieuwenhoven

**Affiliations:** 1 Department of Physiology, Cardiovascular Research Institute Maastricht (CARIM), Maastricht University, 6200 MD Maastricht, The Netherlands; 2 Institute for Technology-Inspired Regenerative Medicine (MERLN), Maastricht University, 6200 MD Maastricht, The Netherlands; 3 MosaMeat, 6229 PM Maastricht, The Netherlands

**Keywords:** stiffness, stretch, cardiac fibroblast, three dimensional

## Abstract

Isolation and culturing of cardiac fibroblasts (CF) induces rapid differentiation toward a myofibroblast phenotype, which is partly mediated by the high substrate stiffness of the culture plates. In the present study, a 3D model of Engineered Heart Matrix (EHM) of physiological stiffness (Youngs modulus ~15 kPa) was developed using primary adult rat CF and a natural hydrogel collagen type 1 matrix. CF were equally distributed, viable and quiescent for at least 13 days in EHM and the baseline gene expression of myofibroblast-markers alfa-smooth muscle actin (Acta2), and connective tissue growth factor (Ctgf) was significantly lower, compared to CF cultured in 2D monolayers. CF baseline gene expression of transforming growth factor-beta1 (Tgfβ1) and brain natriuretic peptide (Nppb) was higher in EHM-fibers compared to the monolayers. EHM stimulation by 10% cyclic stretch (1 Hz) increased the gene expression of Nppb (3.0-fold), Ctgf (2.1-fold) and Tgfβ1 (2.3-fold) after 24 h. Stimulation of EHM with TGFβ1 (1 ng/mL, 24 h) induced Tgfβ1 (1.6-fold) and Ctgf (1.6-fold). In conclusion, culturing CF in EHM of physiological stiffness reduced myofibroblast marker gene expression, while the CF response to stretch or TGFβ1 was maintained, indicating that our novel EHM structure provides a good physiological model to study CF function and myofibroblast differentiation.

## 1. Introduction

The cardiac extracellular matrix (ECM) is a network of structural proteins, mostly collagen fibers, which provides structural stability, tensile strength but also alignment cues, biochemical signals and mechanical support to surrounding cells [1,2,3]. Cardiac fibroblasts (CF) are the cells producing the structural and regulating components of the ECM [4,5] and are therefore important for maintaining the integrity of the ECM [6,7]. In response to injury CF become activated, then differentiate to so called myofibroblasts [8,9] showing special morphological and functional characteristics, such as the expression of alpha smooth muscle actin (αSMA, encoded by the ACTA2 gene) [9,10]. Myofibroblasts are key players in cardiac structural remodeling [11,12,13,14], producing excessive collagen, resulting in cardiac fibrosis and increased myocardial stiffness [15,16].

CF can be isolated from the heart and cultured to study their function in vitro. For many years, these studies have been performed in monolayers (defined as two dimensional (2D)-cultures). In this model, cells attach to a flat surface and cell-matrix attachments are restricted to one plane, while in vivo these attachments are present all around the cells [17,18,19]. Two-dimensional culture conditions also limit cell–cell interactions, as cells grow in monolayers [20]. In addition, culturing CF on hard plastic cell culture plates promotes CF activation and differentiation into myofibroblasts [21,22]. Stiffness is an important stimulus in this process [23], shown in cardiac fibroblasts [24] but also other types of fibroblasts [25,26,27]. Therefore, several groups started generating 3D culture systems to allow in vitro investigation of the cell–matrix interactions in a more physiologically relevant environment [17,18,19].

The aim of the present study was the development of a 3D cell culture model of engineered heart matrix (EHM) of physiological stiffness and to compare CF function in EHM structures vs. 2D monolayers and their response to TGFβ1 and mechanical stimulation. To this purpose, primary adult rat CF were cultured in a natural collagen type 1 hydrogel and stiffness was determined. To gain insight into the CF activation state, we measured gene expression of genes related to CF activation and myofibroblast differentiation: alfa-smooth muscle actin (Acta2) [28], connective tissue growth factor (Ctgf) [29] transforming growth factor beta 1 (TGFβ1) [30,31] and brain natriuretic peptide (Nppb) [32]. Baseline gene expression levels of these genes in EHM cultures were compared with CF cultured in 2D monolayers both on cell culture plastic and on Bioflex silicone bottom plates. Finally, EHM responses to cyclic stretch (10%, 1 Hz) and TGFβ1, both established stimuli for CF-activation, were determined.

## 2. Materials and Methods

### 2.1. Isolation and Culturing of CF

CF were isolated from cardiac ventricles (combined left and right) of adult surplus rats from any age, weight, sex or breed (*n* = 48). Most of the rats used were either from the Lewis or Wistar strain and aged between 5 and 52 weeks. Rat cardiac ventricular fibroblasts were isolated and cultures as previously described [33,34,35] in Dulbecco’s modified eagles medium (DMEM; no. 22320, Gibco, Thermo Fisher Scientific, Waltham, MA, USA) supplemented with 10% (*v*/*v*) fetal bovine serum (FBS, Gibco), gentamicin (50 µg/mL, Gibco), 1% (*v*/*v*) Insulin-Transferrin-Selenium-Sodium Pyruvate (ITS-A, Gibco), basic fibroblast growth factor (1 ng/mL, Gibco) and vitamin C (500 uM, Sigma Aldrich, Saint Louis, MO, USA) (CF growth medium, CFGM) on standard cell culture flasks (Cellstar, Greiner Bio-One, Frickenhausen, Germany). The vast majority of these cells are fibroblast-like cells and these primary fibroblasts were used between passage 1 and 3. Experiments were performed with approval of the Animal Ethical Committee of Maastricht University (DEC-2007-116, 31 July 2007) and conformed to the national legislation for the protection of animals used for scientific purposes.

### 2.2. Assembly of the Ring Formation Molds

**Figure 1:**
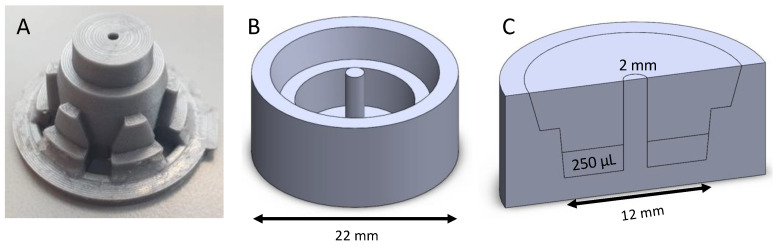
Silicone mold design. Custom made 3D-printed casts to provide the shape for the silicone mold (**A**); The silicone mold had a diameter of 22 mm (**B**); and a centrally placed post 2 mm (**C**). The initial gel volume was 250 μL and after gelation 500 μL of the medium was added on top.

Sylgard-184 silicone elastomer base and curing agent (Dow Chemical, Midland, MI, USA) were mixed together and poured into a well of a 12-well culture plate. Custom made 3D-printed casts (Mosa Meat, Maastricht, The Netherlands) were used to provide the shape with an outer diameter of 22 mm. An area with a 12 mm diameter was created to load 250 uL of gel. A 2 mm central pole created the ring shape (Figure 1). The mold was allowed to cure at room temperature for 3 days after which the 3D-printed casts were removed. The custom-made molds were cleaned and sterilized by autoclavation.

### 2.3. Collagen Hydrogel

Collagen type 1 from rat tail (5 mg/mL, Ibidi, Gräfelfing, Germany) was diluted with sterile water, 10× DMEM, 20× NaHCO_3_ and CF until the desired final collagen concentration (between 1 and 3 mg collagen/mL) and cell density, with the final gel containing 1× DMEM.

### 2.4. Engineered Heart Matrix Ring (EHM-Ring) Formation

CF were grown in CFGM on Cellstar cell culture flasks to about 80–90% confluency, detached using trypsin-EDTA (Gibco) and taken up in CFGM. Cells were centrifuged at 1500 rpm for 5 min and then resuspended in CFGM to reach a concentration of 10 million cells/mL. The cells were diluted to 1 million cells/mL into the hydrogel mixture. The hydrogel –cell mixture (250 μL) was reverse pipetted into the molds and put into a 5% CO_2_ incubator at 37 °C. After approximately 1 h, to polymerize the gel, 500 μL of CF maintenance medium (CFMM, consisting of CFGM with 1% FBS) was added on top of the gels.

### 2.5. Engineered Heart Matrix Fiber (EHM Fiber; Flexcell Tissue Train)

**Figure 2:**
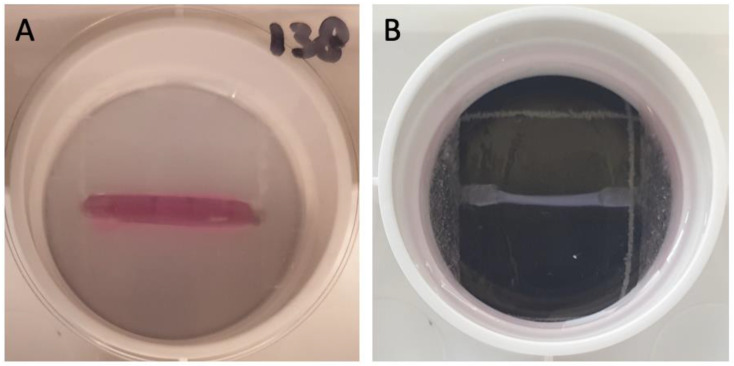
Images of EHM fibers in Flexcell Tissue Train culture plates. Image (**A**) shows the (pink) gel after pipetting in the 6 well Flexcell Tissue Train culture plate, still in the Trough Loader (white bottom) providing the mold for the gel; Image (**B**) shows the EHM fiber between the two anchors after polymerization of the gel. The black and white grid underneath represents 3 cm by 3 cm. The diameter of the well was 3.5 cm.

Using the optimized EHM ring protocol longitudinal EHM fibers were formed in the Flexcell Tissue Train system. This was achieved by pipetting 200 μL of the hydrogel–cell mixture between the two collagen-1-coated anchors of the Tissue Train culture plates (6-well plates, Flexcell Dunn Labortechnik, Asbach, Germany) atop of the Trough Loaders (Figure 2). After approximately 1 hour of polymerization of the gel, 4 mL of CFGM was added on top of the gels. The next day the CFGM was replaced by CFMM. Subsequently, gels were subjected to cyclic stretch (10%, 1 Hz), (Flexcell FX-5000 strain unit, Dunn Labortechnik) for 4 h or 24 h. Control, non-stretched gels were subjected to identical conditions but without stretch being applied.

### 2.6. Measurement of EHM Stiffness

To determine the stiffness of the EHM ring, mechanical analysis was performed using an Electroforce-3200 Series III multiaxial tensile tester (TA Instruments, Asse, Belgium) combined with a 1000 gf (10 N) load cell (1 kg/cm^2^), as previously described [36,37]. Test setups and data acquisition were directed through the WinTest 7 operational software (TA Instruments). The displacement mode of loading materials was controlled through vertical, axial movement with a motorized extension arm (DispE, −40/40 mm). The EHM ring was locked into place using a custom-made Radial Tensile Strength tool (MERLN, Institute for Technology-Inspired Regenerative Medicine, Maastricht University, Maastricht, The Netherlands), based on previous research [38,39].

Uniaxial displacement was applied at a rate of 1% strain per second (0.04 mm/sec.) until EHM ring failure. Load (N) and displacement (mm) were recorded over time at a rate of 20 points/second. The obtained raw datasets were processed in Microsoft Excel and converted to a dataset representing exerted stress (σ, kPa) over strain (ε, %). Noise within the stress–strain curve was reduced using a moving average analysis, where the interval average was set at 20 points to equalize all measured data points per second. Young’s moduli were calculated from the slope of 15% strain residing within the elastic region of the stress/strain curve.

### 2.7. Histology and Immunohistochemistry (IHC)

EHM rings or fibers were washed in PBS (Thermo Fisher), fixed in 4% paraformaldehyde (Klinipath) for 20 min, stained in eosin (J.T.Baker) for 1 h and stored overnight in 70% ethanol (Sigma Aldrich). Finally, the EHM ring or fiber was embedded into paraffin wax. Sections of 5 µm were cut using a rotary microtome.

Hematoxylin and eosin (H&E) staining was performed to gain insight in the cellular distribution and the extracellular matrix (ECM) structure. After rehydration, the slides were placed in Hematoxylin (5 min), washed with running tap water (10 min), placed in eosin (1 min) and washed with demi water. Dehydration steps were performed and the slides were closed with Entallan. Images were obtained using a Leica Microscope (5×, 10× or 20× magnification).

Vimentin and CNA35 IHC were performed to visualize vimentin-positive cells, implicated on being CF and/or collagen matrix. Slides were stained with vimentin antibody (1/150 dilution, ab92547, Abcam, Cambridge, UK) followed by appropriate secondary antibody (1/500 dilution) or adding CNA35 (1/100 dilution) [40,41]. Sections were further incubated in 4′,6′-diamidino-2-phenylindole (DAPI)-containing mounting medium (Vector Laboratories, Burlingame, USA) to stain nuclei. Images were obtained using a Leica fluorescent microscope (40× magnification) or a Leica SPE confocal microscope (63× magnification).

### 2.8. Gene Expression Analysis

Total RNA was isolated from cells using an RNA isolation kit (Omega Biotek, Norcross, GA, USA) and reversed transcribed into cDNA using the iScript cDNA synthesis kit (Biorad, Hercules, CA, USA) according to the manufacturer’s instructions and previously described [32]. Real-time PCR was performed on an CFX96 Touch Real-Time PCR Detection System using iQ SYBR-Green Supermix (Biorad). Gene expression levels of Alpha-smooth muscle actin (Acta2), Connective tissue growth factor (Ctgf), Transforming growth factor, beta 1 (Tgfβ1) and Brain Natriuretic Peptide (Nppb) were normalized using the housekeeping gene Cyclophilin-A (Cyclo), and their relative expression was calculated using the comparative threshold cycle (Ct) method by calculating 2^ΔCt^ (e.g., 2^(Cyclophilin Ct–Nppb Ct)^). The gene expression values were multiplied by 1000 (formula 1000 × 2^ΔCt^), to enhance readability. The sequences of the specific primers used are provided below (Table 1).

### 2.9. Statistics

Data are presented as average, average ± standard deviation or individual data points (indicating separate CF isolations) and were analyzed with the Wilcoxon matched pairs test, the Kruskal–Wallis test, or the Dunn posthoc test where appropriate (Graphpad PRISM V9). Differences were considered statistically significant when *p* < 0.05.

## 3. Results

### 3.1. Optimizing CF Cell Density in EHM Rings

**Figure 3:**
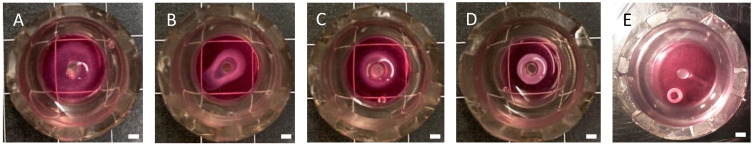
Images of EHM rings taken 24 h after casting the gel using different cell densities (**A**–**D**): 100; 200; 400 and 2000 CF cells/μL using a 1 mg/mL collagen concentration. The images show the top view of the EHM-rings within the clear silicone mold on a black background with white grid (1 cm by 1 cm). Surrounding the central pole is a light pink circle, most left vaguely showing, becoming clearer with increasing the cell density; The final image (**E**) shows the EHM-ring (8000 cells/µL, 1 mg/mL collagen concentration) after 7 days of culturing, next to the central pole. The EHM ring has been removed from the central pole manually to enhance visibility. Bars represent 2 mm.

Different CF cell densities were cultured in EHM rings using a hydrogel collagen concentration of 1 mg/mL in CFMM. EHM ring formation (compaction) was clearly less in the two lower cell densities (Figure 3). The two higher cell densities showed considerably more compaction leading to an EHM ring with a wall thickness of approximately 1.3 mm when using 2000 cells/μL, while using 400 cells/μL led to a wall thickness of approximately 2.1 mm.

### 3.2. Optimizing the EHM Initial Hydrogel Collagen Concentration

**Figure 4:**
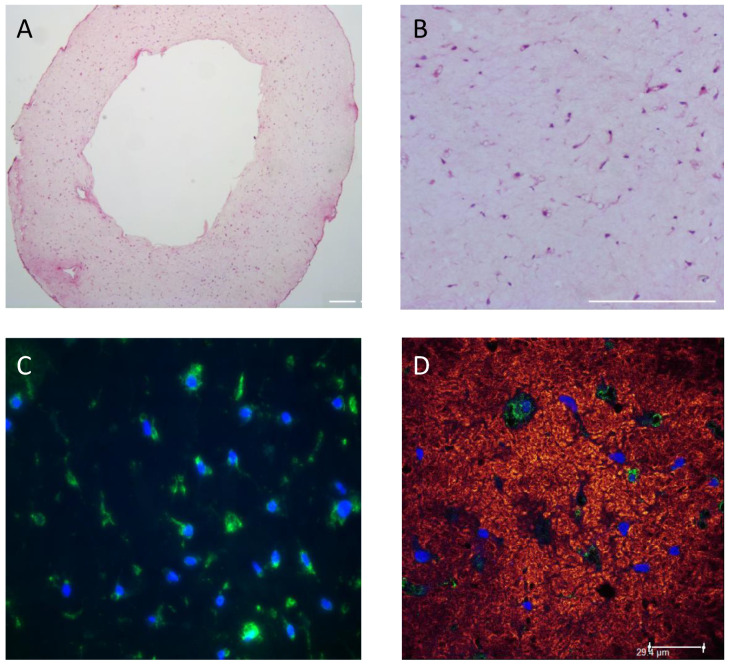
Histological and immunohistological analysis of EHM-ring sections. HE staining of an EHM ring section showing even distribution of the cells throughout the EHM ring structure. EHM ring was formed using 1.5 mg/mL collagen concentration and 1000 CF cells/μL gel and was fixed and stained 24 h after casting. Darker dots implicate CF. Magnification 5× (**A**); or 20× (**B**). Bars represent 200 µm. Vimentin (green) and DAPI (blue) staining (**C**); and Z-stack image of CNA35 (red) and Vimentin (green) staining nuclei are blue (DAPI) (**D**) (bar represents 29.4 μm).

Two different initial hydrogel collagen concentrations (1 and 3 mg/mL) were used to vary the stiffness of the EHM ring [42]. Gel compaction of the 3 mg/mL collagen EHM-ring was reduced resulting in an increased wall thickness compared to EHM ring of 1 mg/mL collagen (4.9 ± 0.9 mm vs 1.8 ± 0.6 mm, *n* = 3) (Supplementary Figure S1). This is reflected by the HE staining (Supplementary Figure S1) where the cell density was lower in the 3 mg/mL collagen compared to the 1 mg/mL collagen EHM ring (65 ± 5 vs 205 ± 25 CF/mm^2^, *n* = 2). RNA isolation from the 3 mg/mL collagen EHM ring resulted in a lower RNA yield when compared to the 1 mg/mL collagen EHM ring (Supplementary Figure S2). Moreover, qPCR analyses revealed increased mRNA expression of Acta2 and Ctgf in the 3 mg/mL collagen EHM-ring, indicating processes towards myofibroblast differentiation, while there was no effect on Tgfβ1 mRNA expression (Supplementary Figure S2). Based on these results, an optimal collagen concentration of 1.5 mg/mL was chosen for further experiments. Using these conditions, we cultured EHM rings for up to 13 days with an average RNA yield of 9.6 pg/cell, measured at day 1, 6, 10 and 13, indicating consistency in viable and quiescent cells over the culturing period. Both histological and immunohistological analysis showed even CF distribution and structural alignment within the EHM structure (Figure 4).

### 3.3. EHM Stiffness

**Figure 5:**
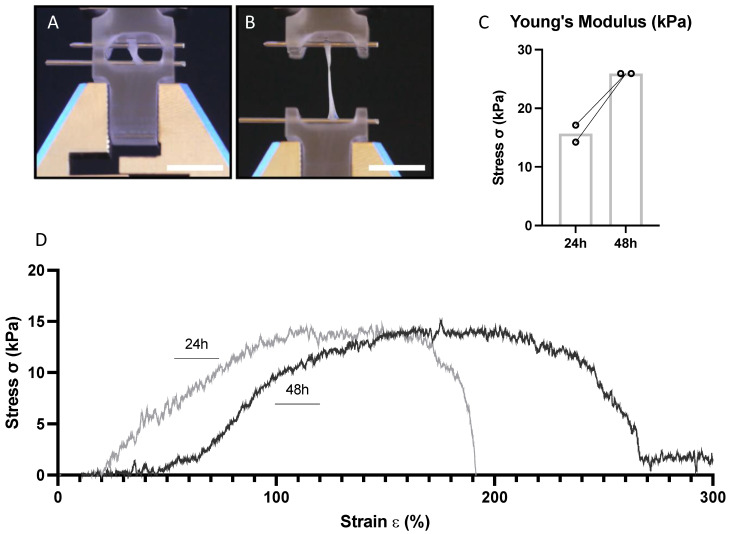
Stiffness measurements of the EHM ring (1.5 mg/mL collagen concentration; 1.000 cells/μL gel) at two different timepoints (24 h and 48 h). Images of tensile analysis at 0% elongation (**A**) and at elongation at break; (**B**). Bar represents 10 mm. Tensile stiffness displayed as Young’s Modulus (*n* = 2); (**C**); Stress–strain (σ/ε) curve of the moving average (20 points/strain value) (*n* = 1) after 24 h and 48 h (**D**).

EHM stiffness was measured at two different timepoints: 24 h and 48 h after casting the ring structures. The EHM ring was locked into place using a custom-made Radial Tensile Strength tool at 0% elongation (Figure 5A) and elongation at break (Figure 5B). After 24 h the average Young’s modulus was 15 kPa and increased to 25 kPa after 48 h (Figure 5C). The stress–strain (σ/ε) curve of the moving average showed the elastic region, followed by the plastic region and breaking point (Figure 5D). These results indicate that the stiffness of our EHM rings was in the same order of magnitude as the passive stiffness of myocardial tissue in vivo, which has been described to be around 10 kPa [43,44,45]. For comparison, the Bioflex plates have been estimated to have a stiffness of ~1000 kPa and plastic culture plates have a stiffness in the gigapascal range [45].

### 3.4. Baseline CF Gene Expression in 3D (EHM) and 2D (Monolayer) Culturse

**Figure 6:**
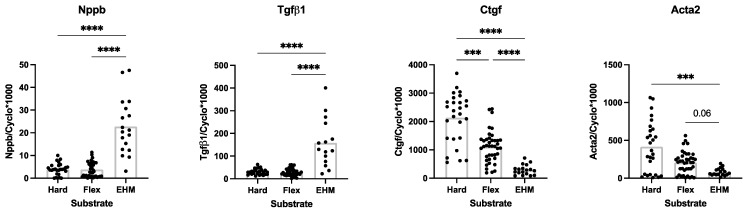
Baseline gene expression levels of Nppb, Tgfβ1, Ctgf and Acta2 in CF cultured on the different substrates: 2D monolayer on hard plastic cell culture plates (Hard), 2D monolayer on Bioflex cell culture plates (Flex) and 3D EHM fibers (EHM). Data are presented as relative mRNA levels normalized to house-keeping gene cyclophylin (*n* = 15–40). *** *p* < 0.001; **** *p* < 0.0001. Bar indicates mean.

Gene expression of Nppb and Tgfβ1, was higher in EHM compared to both regular hard plastic cell culture plates and Bioflex cell culture plates (Figure 6). The opposite was true for the gene expression of Ctgf and Acta2, where the expression was much lower in EHM fibers compared to both hard plastic and Bioflex cell culture plates (Figure 6). The mRNA expression of Ctgf and Acta2 decreased with decreasing the stiffness of the culture substrate.

### 3.5. Stretch of EHM Fibers in the Flexcell Tissue Train System

**Figure 7:**
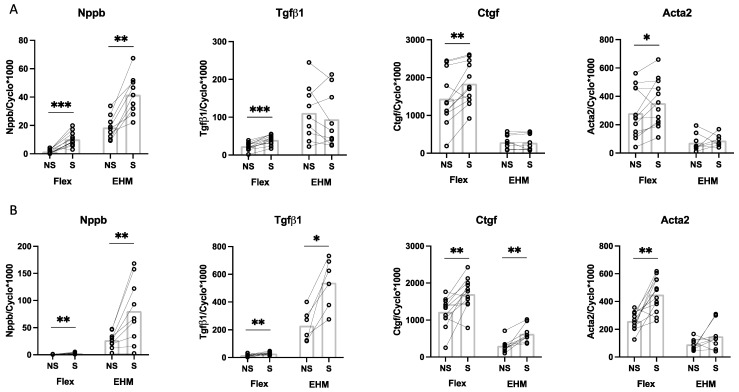
The effect of 4 h (**A**); and 24 h (**B**) cyclic stretch (10%, 1 Hz) on gene expression in CF cultured on 2D monolayer Bioflex plates (Flex) (data from [32]) or 3D EHM fibers (EHM) conditions. Presented are the relative gene expression levels of Nppb, Tgfβ1, Ctgf and Acta2 in 2D (*n* = 5–12) and 3D (*n* = 6–9) in stretch (S) and non-stretch (NS) conditions. * *p* < 0.05; ** *p* < 0.01; *** *p* < 0.001. Bar indicates mean.

Rat CF in EHM fibers exposed to 4 h cyclic stretch (10%, 1 Hz) showed a significantly higher gene expression (2.2-fold) of Nppb compared to non-stretched controls (Figure 7A, EHM). Since similar experiments have previously been performed in 2D CF monolayers on Bioflex plates [32], the effects of cyclic stretch were also compared between Bioflex plates and EHM fibers. No effect of 4 h cyclic stretch (10%, 1 Hz) was found on the gene expression of Tgfβ1, Ctgf and Acta2 in the EHM fibers. In EHM fibers exposed to 24 h of cyclic stretch (10%, 1 Hz) the increase in Nppb remained (3.0-fold). In addition, there was an increased mRNA expression of Ctgf (2.1-fold) and Tgfβ1 (2.3-fold) compared to non-stretched controls (Figure 7B, EHM). Acta2 gene expression showed a 1.6-fold increase after 24h, but this difference did not reach statistical significance. Baseline gene expression differences as indicated in Figure 6 were also seen when comparing the non-stretch conditions of flex plates and EHM culture conditions.

### 3.6. Effect of TGFβ1 Stimulation on CF Gene Expression in 2D (Monolayer) and 3D (EHM) Cultures

**Figure 8:**
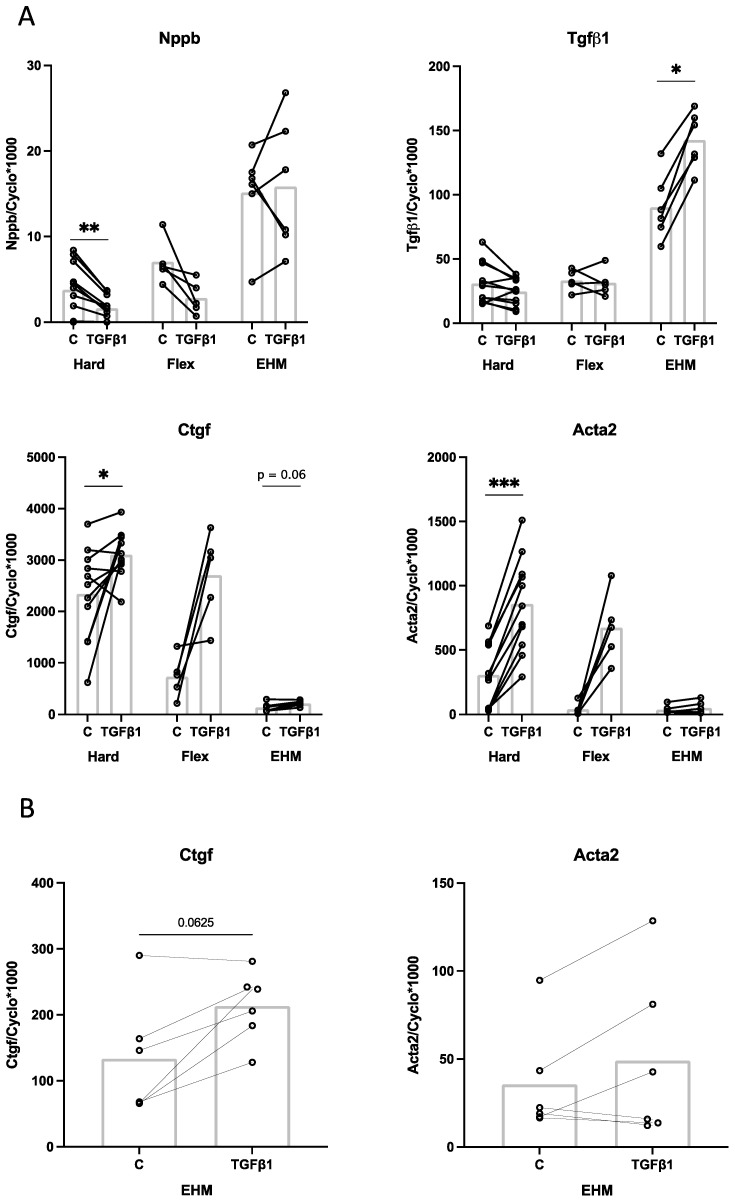
The effect of TGFβ1 stimulation of rat CF for 24 h cultured on different substrates. Presented are the relative gene expression levels of Nppb, Tgfβ1, Ctgf and Acta2 after stimulation with TGFβ1 (TGFβ1) or control (C) conditions for 24 h in 2D hard plastic cell culture plates (Hard, *n* = 11), 2D Bioflex plates (Flex, *n* = 5) and 3D EHM (EHM, *n* = 6) (**A**); Graphs of the relative gene expression levels of Ctgf and Acta2 in EHM were enhanced to improve readability (**B**). * *p* < 0.05; ** *p* < 0.01; *** *p* < 0.001. Bar indicates mean.

TGFβ1-stimulation (1 ng/mL) of CF for 24 h in 2D monolayers on hard plates showed a significant reduction in Nppb gene expression, no effect on Tgfβ1 gene expression and a significant induction of Acta2 and Ctgf gene expression (Figure 8A, Hard). Similar effects of TGFβ1 stimulation on Nppb, Tgfβ1, Ctgf and Acta2 gene expression were observed in 2D monolayers on Bioflex plates (Figure 8A, Flex). Stimulation with TGFβ1 in 3D EHM rings showed no effect on Nppb expression, although as described above Nppb baseline expression levels were higher in the EHM than in the 2D cultures. TGFβ1 stimulation in EHM significantly increased Tgfβ1 gene expression (1.6-fold) (Figure 8A, EHM). In addition, Ctgf showed 1.6-fold increased expression upon TGFβ1 stimulation, although this did not reach statistical significance (Figure 8B). No clear effect of TGFβ1 stimulation on Acta2 was observed in EHM (Figure 8). These results indicate that although the baseline gene expression levels are different in 3D EHM cultures, CF are still capable of responding to TGFβ1 in a similar way as they would in 2D monolayers.

## 4. Discussion

In this study we designed self-assembling EHM rings and EHM fibers composed of rat tail collagen 1 and adult rat ventricular CF, as models for 3D culturing. The stiffness of our EHM rings was ~15 kPa. Comparison of CF gene expression between 2D monolayers and 3D EHM revealed reduced gene expression of Acta2 and Ctgf in EHM, indicating a more quiescent CF state. Cyclic stretch and TGFβ1 stimulation of EHM structures showed CF activation, comparable to 2D cultures of CF. These data indicate that the EHMs provide a more physiological model to study CF function.

### 4.1. Influence of Substrate Stiffness on Baseline CF Gene Expression

Our finding of lower baseline gene expression of Acta2 and Ctgf in 3D EHM cultures compared to 2D monolayers is in line with results from other studies showing that culturing CF on hard plastic cell culture plates promotes myofibroblast differentiation [45,46,47]. Moreover, human CF cultured in low stiffness GelMA gels also showed a reduced expression of ACTA2 [48]. A more quiescent state of fibroblasts cultured in 3D was also found in human fetal lung fibroblasts [49] and tendon fibroblasts [50]. Therefore, both cardiac and non-cardiac CF respond to 3D culturing in a similar way. Taken together, fibroblasts appear to remain in a more quiescent state, i.e., less prone to differentiate into myofibroblasts, when cultured in EHM compared to 2D monolayers. Given the large difference in stiffness between the EHM (~15 kPa) and culture plates (>1000 kPa), it is likely that the quiescent state of CF in EHM, results from the more physiological stiffness of the 3D culture substrates [46,51].

### 4.2. Possible Influence of Culture Medium Differences on Baseline CF Gene Expression

Aside from differences in stiffness between EHM and 2D monolayers, differences due to the use of different culture media cannot be excluded and may affect baseline CF gene expression. The EHM fibers were cultured in CFMM, containing 1% fetal bovine serum (FBS), while the experiments in 2D monolayers were performed in serum-free conditions. FBS is known to have an effect on the expression of many genes [35]. However, for Acta2 it is known that culturing in the presence of serum increases the expression [24,35], while we showed a lower Acta2 expression in our EHM-fibers (CFMM, 1% serum), compared to 2D monolayers, indicating that this lower Acta2 mRNA expression is unlikely to be caused by serum. Galie et al. investigated the effect of serum conditions (5% or 10% FBS) on Tgfβ1 expression in rat cardiac fibroblasts cultured in 3D collagen matrix. Culturing the gels for 24 h resulted in a lower Tgfβ1 mRNA expression in 10% serum compared to 5% serum, measurements during other timepoints (6, 48 and 120 h) showed no difference between 5% or 10% serum on Tgfβ1 mRNA expression [24]. These results implicate that the higher Tgfβ1 mRNA expression we showed in the EHM fibers was most likely not caused by the 1% serum used in those culture conditions. It is important to note here that our FBS percentage of 1% was much lower than those used by Galie and colleagues [24].

### 4.3. Effect of Cyclic Stretch and TGFβ1 on CF Gene Expression

Cyclic stretch for 24 h increased the gene expression of Nppb, Tgfβ1 and Ctgf in both the 2D monolayer and 3D EHM. Acta2 expression was significantly increased in stretched 2D monolayers, but the 1.6-fold increase in EHM was not statistically significant (*p* = 0.20). This lack of significance was most likely caused by low statistical power and large individual variation. Taken together, these data indicate that stretch initiates fibroblast activation, ultimately leading to myofibroblast differentiation. Our results showing an increased mRNA expression of Acta2 in EHM after exposure to cyclic stretch are supported by previous research in NIH 3T3 fibroblasts [52] and marrow-derived progenitor cells [53], using a collagen and fibrin 3D construct, respectively. Primary murine dermal fibroblasts within a collagen 3D construct exposed to 24 h cyclic stretch support our results in showing an increased expression of Tgfβ1 and Ctgf [54]. However, rat cardiac fibroblast-seeded collagen gels exposed to 5% cyclic strain showed a decrease in Tgfβ1 mRNA expression compared to controls [55]. Differences could be attributed to the type of mechanical stimulation applied, compression vs. cyclic stretch. We have previously shown Nppb as being a sensitive marker for stretch in 2D monolayers [32]; this statement was reinforced when a similar strong increase in Nppb gene expression in EHM fibers, both after 4 h and 24 h of cyclic stretch, was shown.

Gene expression of Nppb and Tgfβ1 in EHM was higher in both stretch and non-stretch conditions compared to the 2D monolayers, in both 4 and 24 h conditions. The opposite was true for the expression of Acta2 and Ctgf, suggesting a quiescent state, which has previously been shown [48]. Although the expression of Nppb and Tgfβ1 in non-stretch conditions was higher, CF in EHM were still able to respond to the stimulus of cyclic stretch by even further increasing the expression of Nppb and Tgfβ1, an interesting finding which merits further investigation.

Stimulation of EHM rings with TGFβ1 showed increased gene expression of Tgfβ1 and Ctgf similar as seen in the 2D cultures. No effect of TGFβ1 stimulation on Acta2 and Nppb expression was found. Our results of increased Ctgf expression after TGFβ1 stimulation are supported by previous research in human cardiac fibroblasts [56], adult rabbit cardiac fibroblasts [29] and human lung fibroblasts [57]. TGFβ1-induced Tgfβ1 expression in CF is something we [29] and others [58] have shown previously. TGFβ1 is a well-known stimulus for myofibroblast differentiation [59,60,61], and our finding of TGFβ1–induced increase in Acta2 mRNA expression in 2D cultures therefore fits into the literature [35,62]. This is further supported by Bracco Gartner et al. [48] in 3D cultures. By contrast, our EHM cultures did not show a clear TGFβ1-mediated induction of Acta2. Possibly this lack of effect indicates that the cells are less prone to TGFβ1-induced myofibroblast differentiation when cultured in EHM. Another possible explanation could be the high baseline expression of Nppb in EHM cultures. We have previously shown that BNP inhibits the TGFβ1-induced Acta2 expression [32]. It could be that the high baseline expression of Nppb translates to high levels of BNP within the culture media, inhibiting the TGFβ1-induced Acta2 expression in EHM, hence the lack of effect we see here. Future research is necessary to investigate the role of Nppb within EHM culture. Taken together, CF remain quiescent in EHM, but exhibit a clear response after stimulation with stretch or TGFβ1.

In the present study, we described the development of 3D engineered heart matrix (EHM) ring and fiber structures, with viable and quiescent CF embedded in a type1 collagen gel of physiological stiffness. Markers of CF differentiation toward myofibroblasts were lower in EHM, compared to 2D cultures, while CF activation by stretch or TGFβ1 was maintained, indicating that these EHM structures are a good model to study the process of CF activation and differentiation toward myofibroblasts.

## Figures and Tables

**Table 1:** Gene specific primer sequences used for real-time qPCR.

Gene	Forward Primer	Reverse Primer
Alpha-Smooth muscle actin (Acta2)	AAGGCCAACCGGGAGAAAAT	AGTCCAGCACAATACCAGTTGT
Connective tissue growth factor (Ctgf)	CACAGAGTGGAGCGCCTGTTC	GATGCACTTTTTGCCCTTCTTAATG
Transforming growth factor, beta 1 (Tgfβ1)	GCACCATCCATGACATGAAC	GCTGAAGCAGTAGTTGGTATC
Brain Natriuretic Peptide (Nppb)	AGACAGCTCTCAAAGGACCA	CTATCTTCTGCCCAAAGCAG
Cyclophilin-A (Cyclo)	CAAATGCTGGACCAAACACAA	TTCACCTTCCCAAAGACCACAT

## Data Availability

The data presented in this study are available from the corresponding author upon request.

## Supplementary Materials

The following supporting information can be downloaded at: https://www.mdpi.com/article/10.3390/bioengineering9100551/s1, Figure S1: Effect of initial collagen concentration on EHM compaction. Figure S2: Effect of initial collagen concentration on RNA yield per cell and gene expression of Acta2, Ctgf and Tgfβ1.
