# Supplementary material for: Culturing of Cardiac Fibroblasts in Engineered Heart Matrix Reduces Myofibroblast Differentiation but Maintains Their Response to Cyclic Stretch and Transforming Growth Factor β1
Source: Bioengineering (Basel). 2022 Oct 14;9(10):551. doi: 10.3390/bioengineering9100551 (PMC9598692; doi:10.3390/bioengineering9100551)

Supplemental figures

Culturing of cardiac fibroblasts in Engineered Heart Matrix reduces myofibroblast differentiation but maintains their response to cyclic stretch and transforming growth factor β1

Meike C. Ploeg [1], Chantal Munts [1], Tayeba Seddiqi[1], Tim J.L. ten Brink[2], Jonathan Breemhaar[3], Lorenzo Moroni[2], Frits. W. Prinzen[1] and Frans. A. van Nieuwenhoven[1]*

[1]Department of Physiology, Cardiovascular Research Institute Maastricht (CARIM), Maastricht University, the Netherlands, [2]MERLN, Institute for Technology-Inspired Regenerative Medicine, Maastricht University, the Netherlands, [3]MosaMeat, Maastricht, the Netherlands.

*Correspondence: f.vannieuwenhoven@maastrichtuniversity.nl

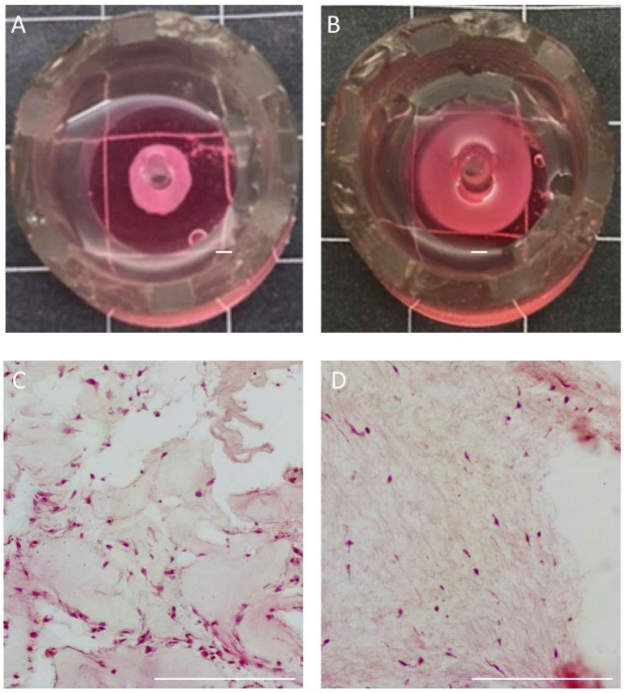

Supplemental Figure S1. Effect of initial collagen concentration on EHM compaction. EHM-rings using 1 mg/ml (A) or 3 mg/ml (B) initial collagen concentrations (2000 cells/µL) in 250 uL gel after 24 h. Images are taken on the black and white grid of 1 x 1 cm. Shown is the silicone mold, the central pole containing the ring in light pink tones, surrounded by CFMM. Bars represent 2 mm. HE staining of the EHM-ring after 24 h using 1 mg/ml (C) and 3 mg/ml (D) initial collagen concentrations (2000 cells/µL in 250 uL gel) showing the cell density difference between the two collagen concentrations. Magnification 20x. Bars represent 200 µm.

Supplemental Figure S2. Effect of initial collagen concentration (1 or 3 mg/ml) on RNA yield per cell (pg/cell) and gene expression of Acta2, Ctgf and Tgfβ1 of the EHM-rings after 24 h of culture (2000 cells/µL in 250 µL gel) (n=2). Bar represents mean.

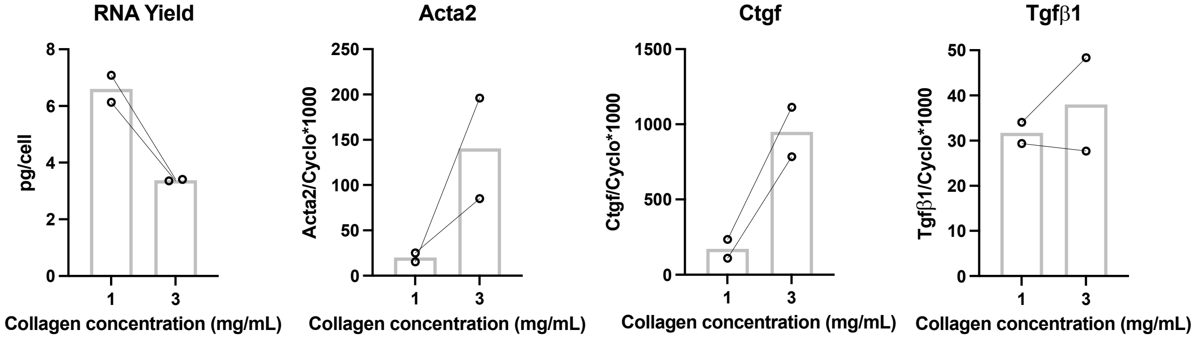

Supplement: Supplementary file 1 [file bioengineering-09-00551-s001.zip › bioengineering-1933982-supplementary.pdf]
